# Supraphysiological doses of performance enhancing anabolic-androgenic steroids exert direct toxic effects on neuron-like cells

**DOI:** 10.3389/fncel.2013.00069

**Published:** 2013-05-09

**Authors:** John R. Basile, Nada O. Binmadi, Hua Zhou, Ying-Hua Yang, Antonio Paoli, Patrizia Proia

**Affiliations:** ^1^Department of Oncology and Diagnostic Sciences, University of Maryland Dental SchoolBaltimore, MD, USA; ^2^Marlene and Stuart Greenebaum Cancer Center, University of MarylandBaltimore, MD, USA; ^3^Department of Oral Basic and Clinical Sciences, King Abdulaziz UniversityJeddah, Saudi Arabia; ^4^Department of Biomedical Sciences, University of PadovaPadova, Italy; ^5^Department of Sports Science (DISMOT), University of PalermoPalermo, Italy

**Keywords:** anabolic-androgenic steroids, PC12, neurotoxicity, apoptosis, neuritin

## Abstract

Anabolic-androgenic steroids (AAS) are lipophilic hormones often taken in excessive quantities by athletes and bodybuilders to enhance performance and increase muscle mass. AAS exert well known toxic effects on specific cell and tissue types and organ systems. The attention that androgen abuse has received lately should be used as an opportunity to educate both athletes and the general population regarding their adverse effects. Among numerous commercially available steroid hormones, very few have been specifically tested for direct neurotoxicity. We evaluated the effects of supraphysiological doses of methandienone and 17-α-methyltestosterone on sympathetic-like neuron cells. Vitality and apoptotic effects were analyzed, and immunofluorescence staining and western blot performed. In this study, we demonstrate that exposure of supraphysiological doses of methandienone and 17-α-methyltestosterone are toxic to the neuron-like differentiated pheochromocytoma cell line PC12, as confirmed by toxicity on neurite networks responding to nerve growth factor and the modulation of the survival and apoptosis-related proteins ERK, caspase-3, poly (ADP-ribose) polymerase and heat-shock protein 90. We observe, in contrast to some previous reports but in accordance with others, expression of the androgen receptor (AR) in neuron-like cells, which when inhibited mitigated the toxic effects of AAS tested, suggesting that the AR could be binding these steroid hormones to induce genomic effects. We also note elevated transcription of neuritin in treated cells, a neurotropic factor likely expressed in an attempt to resist neurotoxicity. Taken together, these results demonstrate that supraphysiological exposure to the AAS methandienone and 17-α-methyltestosterone exert neurotoxic effects by an increase in the activity of the intrinsic apoptotic pathway and alterations in neurite networks.

## Introduction

Anabolic-androgenic steroids (AAS) are lipophilic hormones, derived from cholesterol, that include in the same family the natural male hormone testosterone, along with the related molecules methandienone, 17-α-methyltestosterone, nandrolone, and androsterone (Orlando et al., 2007). For many years athletes and bodybuilders have been taking AAS in supraphysiological doses in order to attain greatly increased muscle mass, well beyond what can be achieved through natural means (Kouri et al., 1995), without regards to their potentially toxic side effects. Intake of AAS by athletes and others in an attempt to gain strength and improve performance is often associated with toxic effects on the liver, the cardiovascular system and the male and female reproductive systems (Trifunovic et al., 1995; Basaria, 2010). At the physiological level, there are a wide range of effects of AAS as they possess both anabolic, or muscle-building, and androgenic, or masculinizing, properties (Kanayama et al., 2007). For example, while AAS use in women is generally uncommon, this population is highly vulnerable to the masculinizing effects of AAS and any resulting reproductive changes (Malarkey et al., 1991; Gruber and Pope, 2000; Kanayama et al., 2007). For these reasons, since 1999 the committee of the Word Antidoping Agency has been monitoring and regulating the use of AAS, along with similar compounds classified as nutritional supplements (http://www.wada-ama.org, *The 2012 Prohibited List*; and Carmo et al., 2011).

AAS exert their effects in many parts of the body, including the reproductive and endocrine tissues, muscle, bone, hair follicles in the skin, the liver and kidneys, and the hematopoietic, immune and central nervous systems (CNS) (Mooradian et al., 1987). For example, experiments carried out on human umbilical vein endothelial cells to study the consequences of treatment with high concentrations of some AAS have revealed significant anti-proliferative effects, dramatically altered intracellular concentrations of calcium and induction of apoptosis (D'Ascenzo et al., 2007). A major concern regarding AAS is their influence on behavior, which is often correlated with stroke, mood disturbances, and psychotic symptoms (Uzych, 1992; Hall and Chapman, 2005; Santamarina et al., 2008). *In vivo* administration of high doses of androgens has been linked to neurobehavioral changes like hyperexcitability, heightened aggressive behavior, and suicidal tendencies (Tirassa et al., 1997; Thiblin et al., 2000). There is evidence that some of this behavior could be caused by the effects of AAS over synapse formation and function (Penatti et al., 2009, 2011; Oberlander and Henderson, 2012). However, particularly under conditions of high concentrations of testosterone and its metabolites, AAS may cause these changes as a result of functional, morphological and biochemical effects on neurons themselves, culminating in cell death (do Carmo et al., 2012).

Inappropriate activation of apoptosis in neurons has been associated with several neurological illnesses, including Huntington disease and Alzheimer disease (AD) (Varshney and Ehrlich, 2003; Tang et al., 2005). Non-physiological concentrations of AAS have been shown to induce neurotoxicity over a long time course when in the presence of β-amyloid, an important component of AD (Caraci et al., 2011). Most of the CNS effects are of psychiatric origin, and whether or not AAS are toxic to neurons is yet unknown. Orlando et al. studied the effect of some AAS on excitotoxic neuronal death induced by N-methyl-d-aspartate (NMDA) in primary cultures of mouse cortical cells. Under these conditions, testosterone amplified excitotoxic neuronal death at high concentrations (10 μM) (Orlando et al., 2007).

Given that AAS abuse poses a significant public health problem and based upon the previously published data, we investigated the morphological, biochemical and molecular mechanisms leading to changes in neuronal physiology, in particular neuronal cell death, for supraphysiological concentrations of methandienone and 17-α-methyltestosterone, two AAS commonly found for sale on the internet and used for gain muscle mass but less studied than other hormones such as nandrolone and androsterone. Derivates of methandienone and 17-α-methyltestosterone also resist metabolism in the liver and contain modifications that are associated with significant hepatic toxicities (Kuhn, 2002); of particular relevance, C17α-alkylated AAS, such as 17α-methyltestosterone, cannot be aromatized to 17β-estradiol and can also inhibit aromatase activity. Supraphysiological levels of 17α-methyltestosterone may promote diminished aromatization (and thus estrogen levels) by directly inhibiting the activity of this enzyme (Penatti et al., 2011).

There are a number of well characterized *in vitro* models that have been used to examine chemical effects on neurite outgrowth (Radio and Mundy, 2008). We chose to utilize the neuron-like PC12 cell model. PC12 are a line that has been widely employed in neurobiological investigations in order to evaluate the effects of different drugs and potentially neurotoxic compounds on cell morphology and physiology (Fujita et al., 1989; Vaudry et al., 2002; Radio and Mundy, 2008). Following exposure to nerve growth factor (NGF), PC12 cells differentiate into a sympathetic-like neuron and develop extensive neuritic processes (Greene and Rein, 1977), making this line an excellent model for evaluation of environmental compounds on neurite outgrowth and differentiation (Chan and Quik, 1993; Das and Barone, 1999; Lein et al., 2000; Crumpton et al., 2001; Parran et al., 2003).

Here we demonstrate that rat neurons and PC12 cells express the androgen receptor (AR). We describe a reduction in neurite networks and loss of survival signaling and enhanced apoptosis, as evidenced by a decrease in phospho-ERK and an increase in the levels of the active fragment of caspase 3 and cleaved poly (adenosine diphosphate [ADP]-ribose) polymerase (PARP), as well as upregulation and cleavage of heat shock protein (Hsp) 90, occurring in a dose-dependent manner in androgen treated PC12 differentiated in NGF. Many of these observations were noted after long exposures of PC12 to AAS, suggesting a genomic effect, and through the use of hydroxyflutamide we demonstrate that AAS toxicity proceeds directly through the AR, likely altering gene transcription to affect cell survival (Heinlein and Chang, 2002; Nguyen et al., 2007). However, we also observed a short-term increase in neuritin expression, an adaptive reaction seen in neurons in response to injury. Taken together, these results suggest that AAS may exert direct toxic effects on neurons of the CNS in those abusing these substances in athletic training.

## Materials and methods

### Cell culture and hormone treatment

Undifferentiated pheochromocytoma 12 cells (PC12, ATCC, Manassas, VA) were cultured in RPMI-1640 medium containing 10% horse serum and 5% fetal bovine serum (Sigma-Aldrich, St. Louis, MO). For induction of differentiation, cells were grown in 12-well plates on poly-D-lysine (Sigma-Aldrich) at an initial concentration of 1 × 10^5^ cells per square centimeter in a medium supplemented with 200 ng/ml of nerve growth factor (NGF, Promega Corporation, Madison, WI) for 5 days. During this time, cells attached to the substratum and produced a network of neurites. Differentiated PC12 cultures were treated with vehicle (dimethyl ether, DME) or the steroid hormones androsterone, nandrolone, methandienone and 17-α-methyltestosterone (Cerilliant Corporation, Round Rock, Texas) at a concentration of 50–75 μM for times defined as short term (24 h), and long term (48 h) (Duranti et al., 2011), and pre-incubated for 2 h with vehicle control or 10 μM hydroxyflutamide (Sigma-Aldrich, St. Louis, MO) (Nguyen et al., 2007) where indicated. Preliminary experiments verified that DME did not have any effects compared to an evaluation of untreated cells (data not shown).

### Immunohistochemistry

Immunohistochemistry on rat brain slices was performed following the protocol described previously (Basile et al., 2006). The primary antibody was anti-AR (Santa Cruz, CA). Images were acquired using a digital SPOT camera (SPOT Imaging Solutions, Sterling Heights, MI) attached to an inverted Nikon phase-contrast microscope (Nikon Instruments, Melville, NY).

### Immunocytochemistry

Control or methandienone and 17-α-methyltestosterone treated PC12 cells were fixed with 4% paraformaldehyde in PBS for 10 min. on ice and subsequently permeabilized in cold methanol for an additional 5 min. After three washes with PBS, cells were blocked with 5.5% FBS in PBS with 0.1% Triton X-100 for 45 min. at room temperature. The cells were then incubated with AR antibody (Santa Cruz, CA) in 3% BSA/ PBS overnight at 4°C. The next day, cells were washed three times in 0.1% Triton/ PBS and once in PBS and incubated with biotinylated secondary antibody (Dako North America, Carpinteria, CA) for 45 min. Cells were then washed three times in 0.1% Triton/ PBS and then once again in PBS, followed by an incubation in 0.6% H_2_O_2_ for 30 min. at room temperature to quench endogenous peroxidase. Cells were then incubated in strep ABC complex (Dako North America) at room temperature for 30 min, washed and incubated with DAB peroxidase substrate (Vector Laboratories, Youngstown, OH) following manufacturer's instructions. Counterstain was performed in dilute Harris hematoxylin (Sigma-Aldrich). Image acquisition was performed as described for immunohistochemistry (see above).

### Immunofluorescence and analysis of neurite network

1 × 10^5^ PC12 cells per square centimeter growing on poly-D-lysine coated cover slips were differentiated for 5 days in 200 ng/ml of NGF (Promega), followed by treatment with 75 μM of methandienone or 17-α-methyltestosterone (Cerilliant) or equal amounts of DME (as the carrier control). Cells were then fixed with 96% ice-cold ethanol for 10 min. and permeabilized for 5 min. with 0.1% Triton X-100 in PBS. Cells were blocked in 3% fetal bovine serum for 30 min., followed by 1 h incubation in a humidity chamber at room temperature with rabbit polyclonal anti-NF antibody (Cell Signaling Technology, Beverly, MA). The secondary antibody was anti-rabbit IgG conjugated to fluorescein (Sigma Aldrich). The samples were mounted with Vectashield mounting medium containing 4-6-diamino-2-phenyl-indole (DAPI, Vector Laboratories). Morphological analysis and quantification of neurite bearing cells were carried out using an Aperio Scanscope (Aperio Technologies, Vista, CA). Ten randomly separated microscopic fields were observed and the proportion of cells with neurites equal to or greater than the length of one cell body were scored positive for neurite outgrowth, with the final result expressed as a percentage of the total number of cells counted. Neurite extension length was also measured for all identified positive neurite bearing cells per field by tracing the longest length neurite using the Neuron J module of Image J software (NIH, Bethesda, MD, version 1.46c). The value of neurite length in pixels (average maximal neurite length per neurite-bearing cell in 10 fields) was calculated and designated as one experiment. All experiments were repeated at least three times on separate days and data are expressed as mean ± SD.

### Vitality assay

Cell death was evaluated by staining PC12 cells treated for 48 h with 75 μM methandienone, 17-α-methyltestosterone or DME with acridine orange/ethidium bromide mixture (Sigma-Aldrich), each at a concentration of 100 μg/ml in PBS as previously described (Schiera et al., 2007). Cells were observed with a Fluoview FV1000 Confocal Microscope (Olympus).

### Western blot analysis

After the indicated treatment, cells were collected, washed with PBS, and homogenized in lysis buffer (Cell Signaling Technology) supplemented with protease inhibitors (0.5 mM phenylmethylsulfonyl fluoride, 1 μ l/ml aprotinin and leupeptin, Sigma-Aldrich) and phosphatase inhibitors (2 mM NaF and 0.5 mM sodium orthovanadate, Sigma-Aldrich). After centrifugation, protein concentrations were measured using the Bio-Rad protein assay (Bio-Rad Hercules, CA). 15 μg of protein was loaded onto each lane of 12% acrylamide-SDS denaturing gels. After electrophoretic separation, samples were electroblotted onto a PVDF membrane (Immobilon P, Millipore Corp., Billerica, MA). The blotting membrane was blocked with 5% milk and then immunostained with one of the following antibodies: anti-phospho-ERK (Cell Signaling Technology); rabbit polyclonal anti-Hsp90 (Cell Signaling Technology); rabbit polyclonal anti-cleaved caspase 3 (Cell Signaling Technology); rabbit polyclonal anti-PARP (Cell Signaling Technology); anti-GAPDH (Sigma-Aldrich).

### RNA extraction, reverse transcription and real time-PCR analysis

RNA was extracted from treated and untreated PC12 cells using the TRIZOL reagent (Life Technologies, Grand Island, NY) according to the protocol provided by the manufacturer. After the last step of the protocol, the RNA was air-dried and then dissolved in RNAase free water for quantification by spectrophotometer. 1 μg of RNA was used for reverse transcription to cDNA using the AMV reverse-transcriptional system (Promega) in the presence of random hexamers (Invitrogen, Life Technologies). The cDNA was used for quantitative real-time PCR (RT-qPCR) with specific gene primers as follows: Neuritin sense: 5′-gcatctggtgaataatcgctcacg-3′, anti-sense: 5′-actgaaggaggcgacgacaatagc-3′; GAPDH sense: 5′-atcccatcaccatcttccag-3′, anti-sense: 5′-cctgcttcaccaccttcttg-3′. The C_T_ method was used for data analysis of neuritin mRNA expression, estimated in triplicate samples and normalized to GAPDH expression levels.

### Statistical analysis

Student's paired *t* tests were performed on means, and *p* values calculated: ^*^, *p* ≤ 0.05; ^**^, *p* ≤ 0.01.

## Results

### AR is expressed in neuron-like PC12 cells

**Figure 1:**
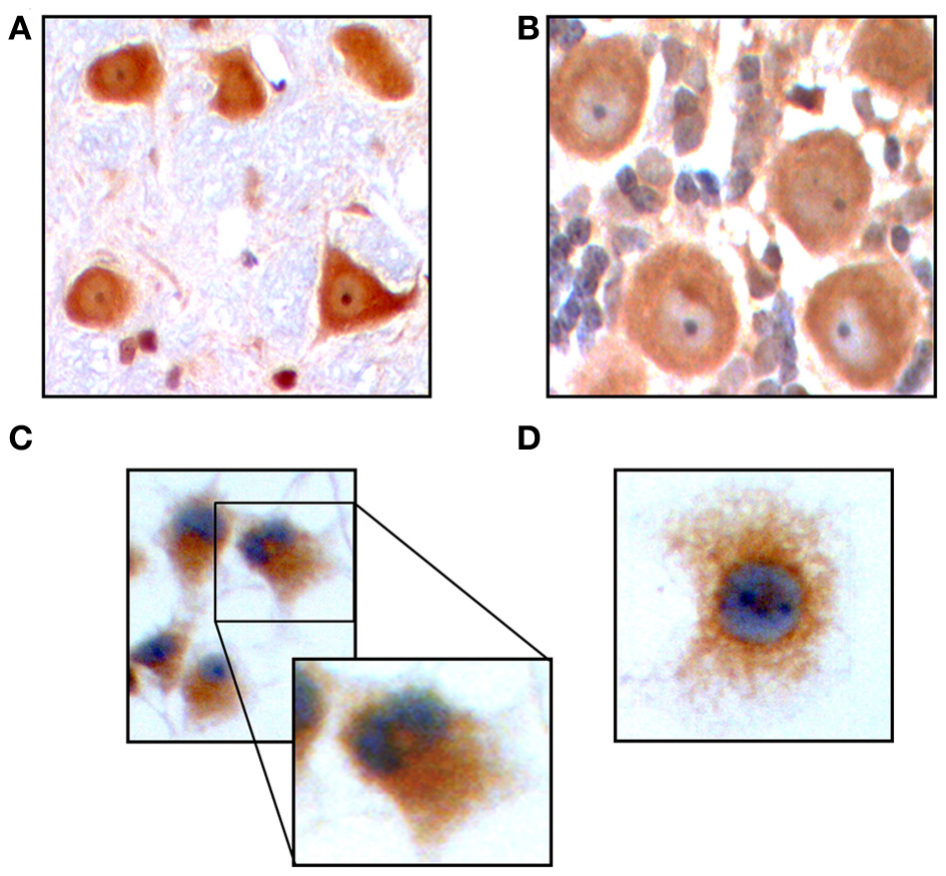
**Immunohistochemistry in Purkinje cells of the rat cerebellum demonstrates expression of AR.** Original magnification 200 X **(A).** AR expression in neurons of the rat brain stem. Original magnification 200 X **(B).** Immunocytochemistry for AR on PC12 in culture. Details of receptor expression are shown in the inset. Original magnification 200 X **(C).** AR expression in PC12 cells. Original magnification 400 X **(D)**.

The AR, a member of the nuclear receptor superfamily of transcription factors, is capable of binding the principal steroidal androgens testosterone and its metabolite 5α-dihydrotestosterone, as well as other AAS, and mediating their effects within the cell (Lee and Chang, 2003). Caraci et al. determined that neurons express this receptor (Caraci et al., 2011), results we confirmed in an immunohistochemical analysis for AR in rat cerebellum (Figure 1A) and brain stem (Figure 1B). To determine if PC12 cells also express this receptor after differentiation and induction of a neuron-like phenotype, we performed immunocytochemistry for AR on NGF treated PC12 in culture. We observed AR expression in the cytoplasm (Figure 1C), which under higher magnification exhibited a peri-nuclear pattern of expression (Figures 1C, *inset*, and 1D), contrary to a prior report (Nguyen et al., 2005) but in accordance with others (Gehlhaus et al., 2007; Meyer et al., 2009). These results indicate that PC12 express the receptor for androgens that could be mediating the pathway for AAS to affect cell physiology.

### Methandienone and 17-α-methyltestosterone inhibit neurite networks

**Figure 2:**
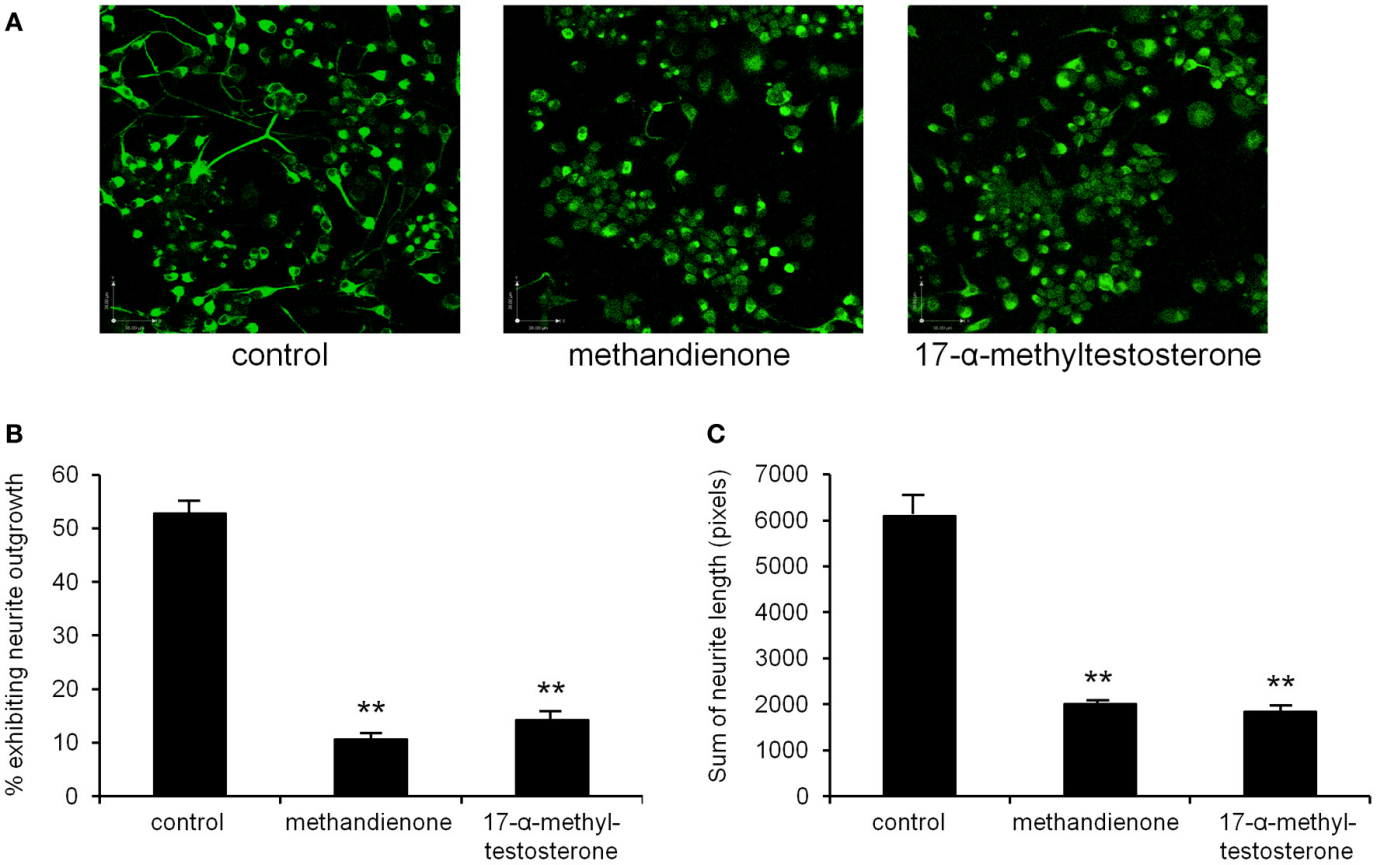
**Immunofluorescence for neurofilament to demonstrate neurite outgrowth in PC12 cells differentiated in NGF in tissue culture, either control treated (*left panel*), treated with 75 μM methandienone (*center panel*) or treated with 75 μM of 17-α-methyltestosterone (*right panel*) (A).** Quantification of neurite outgrowth in the three conditions shown in **(A)**, as determined by cells exhibiting neurites equal to or greater than the length of one cell body, as a percentage of the total number of cells counted (*Y-axis*). Experiments were repeated at least three times and data expressed as mean ± SD (^**^P ≤ 0.01). These AAS demonstrate toxic effects on formed neurite networks in differentiated PC12 **(B).** Average of the sums of neurite length for identified neurite bearing cells in 10 high-power fields (*Y-axis*). Experiments were repeated at least three times and data expressed as mean ± SD (^**^P ≤ 0.01) **(C)**.

Neuron cell cultures are a useful system to study potential deleterious effects of different compounds. In this model, alterations in sprout formation and neurite length are used as a determinant of neurotoxicity (Radio and Mundy, 2008). Since culturing PC12 cells in NGF induces formation of neurites, we used this model to determine the effects of methandienone and 17-α-methyltestosterone on neurons, growing treated cells in control media or media containing 50 or 75 μM of these AAS and observing effects on differentiated PC12 neurite outgrowth. While untreated cells exhibited prominent neurite maintenance (Figure 2A, *left panel*), cells treated with both methandienone (*center panel*) and 17-α-methyltestosterone (*right panel*) lost this phenotype. Quantification of loss of neurite formation is shown in Figure 2B. Both of these AAS also resulted in dramatic reduction of the total length of all neurites observed, relative to vehicle controls (Figure 2C). Taken together, the results suggest that these AAS can exert toxic effects on neuronal networks, inducing neurite loss and neuronal network damage.

### Methandienone and 17-α-methyltestosterone induce death of PC12 cells

**Figure 3:**
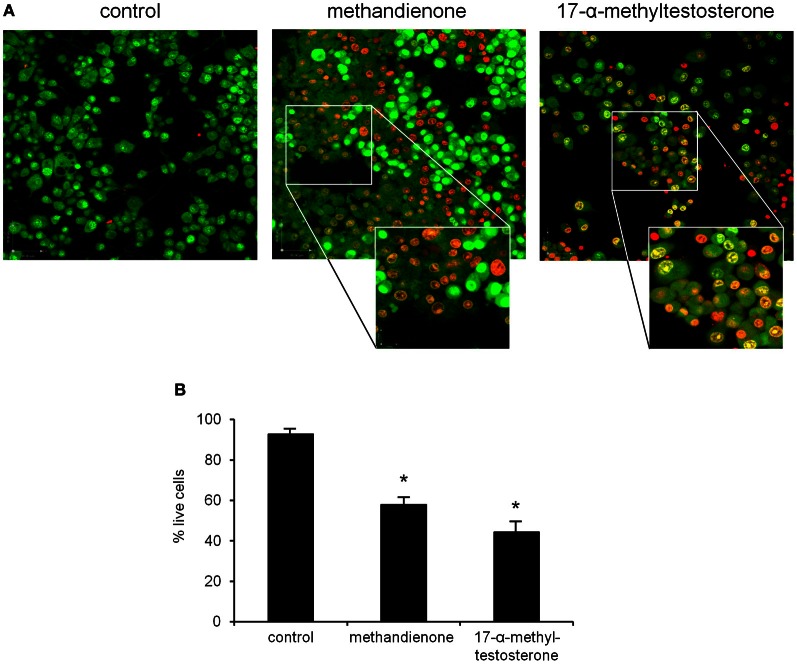
**PC12 cells were control treated (*left panel*) or treated with 75 μM methandienone (*center panel*) or 75 μM 17-α-methyltestosterone (*right panel*) for 48 h and a vitality assay was performed.** Green cells are vital; cells in early cell death are yellow; orange indicates late cell death **(A).** Vitality status expressed as percentage of total live cells counted in 10 high power fields (*Y-axis*) reveals that methandienone and 17-α-methyltestosterone induce cell death in PC12. Experiments were repeated at least three times and data expressed as mean ± SD (^*^P ≤ 0.05) **(B)**.

To further determine toxicity, PC12 were grown in 75 μM of methandienone and 17-α-methyltestosterone and examined for cell death in a vitality assay. In this system, cells are analyzed by immunofluorescence to detect membrane integrity based upon the uptake or exclusion of a dye from the cell. Ethidium bromide (EB) fluoresces red and is only able to pass through the membrane of a dead or dying cell, while acridine orange (AO), which fluoresces green, is a membrane-permeable dye that will stain all cells in the sample. Cells fluorescing yellow are taking up both EB and AO and represent an early stage of cell death, with a more orange color indicating a later stage in the process. We observed that most control treated, differentiated PC12 remained vital (Figure 3A, *left panel*) while cells treated with both methandienone (*center panel*) and 17-α-methyltestosterone (*right panel*) exhibited prominent increases in indicators of early and late cell death (see *insets*). When quantified, we observed slightly more cell death in PC12 growing in the presence of 17-α-methyltestosterone when compared to equal concentrations of methandienone (Figure 3B). These results suggest that both methandienone and 17-α-methyltestosterone are toxic to neurons, inducing death in treated cells.

### AAS induce apoptosis in PC12

**Figure 4:**
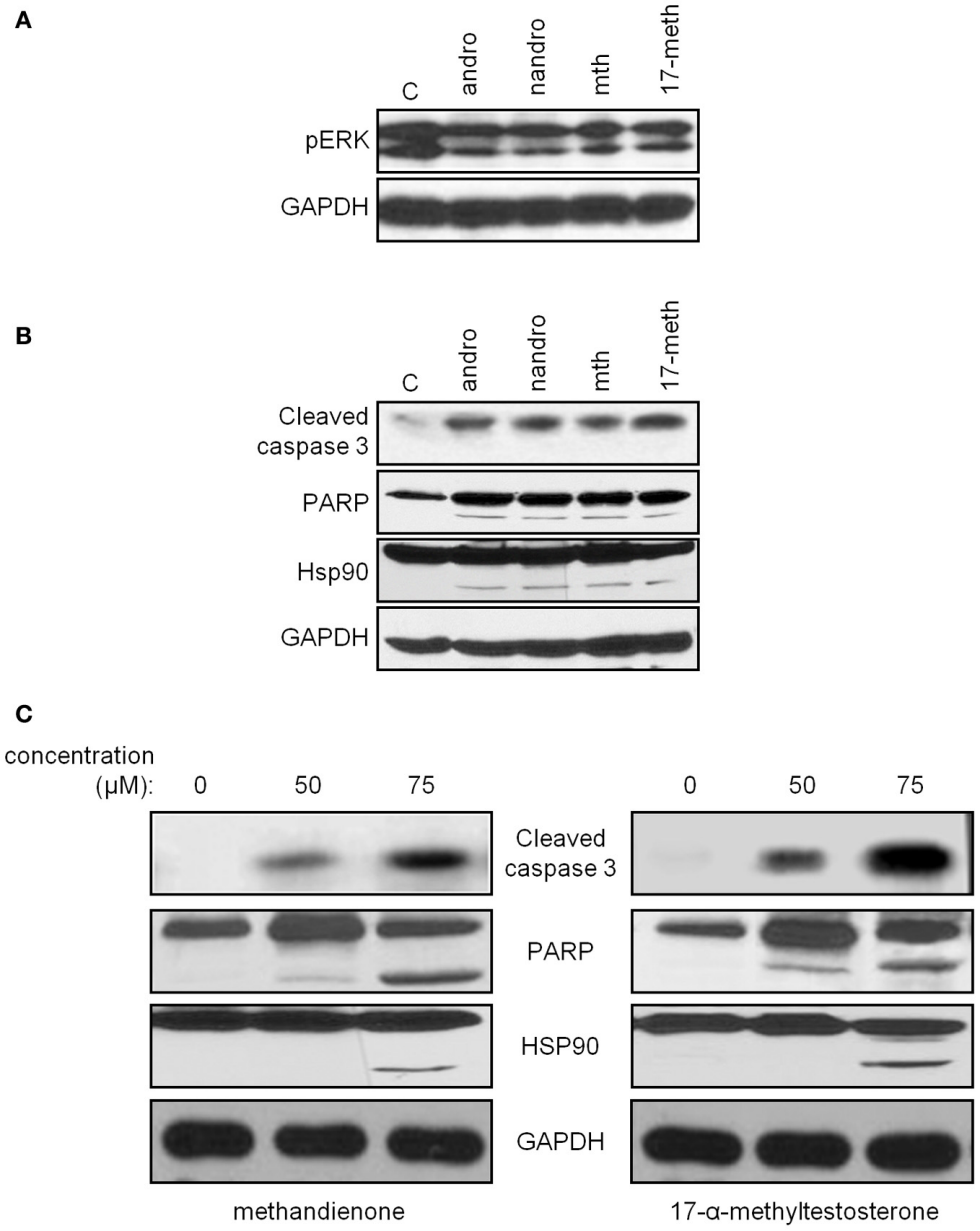
**Immunoblots demonstrate decreasing levels of phospho-ERK (*top panel*) in PC12 treated for 48 h with 75 μM androsterone, nandrolone, methandienone, and 17-α-methyltestosterone, compared to control treated cells **(A)**.** Immunoblots for caspase 3 (*top panel*), PARP (*second panel*), and Hsp90 (*third panel*) in PC12, control treated or treated 48 h with 75 μM androsterone, nandrolone, methandienone, and 17-α-methyltestosterone demonstrate increasing levels of the activated fragment of caspase 3 and cleavage of PARP and Hsp90, indications of apoptosis and cell death **(B)**. PC12 were control treated or treated for 48 h with 50 or 75 μM methandienone (*left column*) or 17-α-methyltestosterone (*right column*). Increasing concentrations of these AAS resulted in appearance of the activated fragment of caspase 3 and cleavage of PARP and Hsp90 in a dose-dependent manner **(C)**. GAPDH was used as the loading control for all blots (*bottom panels*).

To confirm that the cell death we observed was apoptosis, and to compare with other AAS to determine if this could be a general mechanism of toxicity for a variety of androgens, we examined by immunoblot for levels of active, phosphorylated ERK, an indicator of cell survival. The MAPK cascade, and in particular ERK, has been shown to be protective in neuronal cell types, allowing them to survive exposure to pro-apoptotic compounds (Karmarkar et al., 2011). Therefore, decreases in phospho-ERK might indicate progression to cell death. We observed decreases in levels of phosphorylated ERK protein following treatment with androsterone, nandrolone, methandienone and 17-α-methyltestosterone, compared to controls (Figure 4A, *upper panel*). We also looked for the appearance of cleaved and hence active caspase 3 as an indicator of apoptosis, cleaved PARP (a caspase target), and cleavage of heat shock protein (Hsp) 90, a chaperone involved in the normal folding of various polypeptides that has been shown to be degraded in cells undergoing apoptosis and that associates with the AR (Chen et al., 2008, 2009), following treatment with the same AAS. We noted upregulation of the active fragment of caspase 3, an indication of activation of the caspase cascade, occurring in response to all AAS used (Figure 4B, *upper panel*). Additionally, we observed the appearance of cleavage products for PARP and Hsp90 (Figure 4B, *middle panels*), proteins that are downstream targets of caspases in apoptosis. These results suggest that neuronal cell death may contribute to some of the CNS symptoms observed with long-term use of AAS.

Examining methandienone and 17-α-methyltestosterone in more detail, we noted upregulation of the active fragment of caspase 3 occurring in a dose dependent manner following treatment of both of these AAS (Figure 4C, *top panels*), along with the appearance of cleaved PARP and Hsp90 (Figure 4C, *middle panels*). GAPDH was used as a loading control for all blots (*bottom panels*). Similar to the cell death response in the vitality assay, we observed slightly more pronounced indicators of apoptosis in the immunoblot for cells treated with 17-α-methyltestosterone compared to methandienone. Taken together, these results suggest that methandienone and 17-α-methyltestosterone are toxic to neuron-like cells at least in part through activation of apoptosis.

### Methandienone and 17-α-methyltestosterone promote apoptosis through the AR

**Figure 5:**
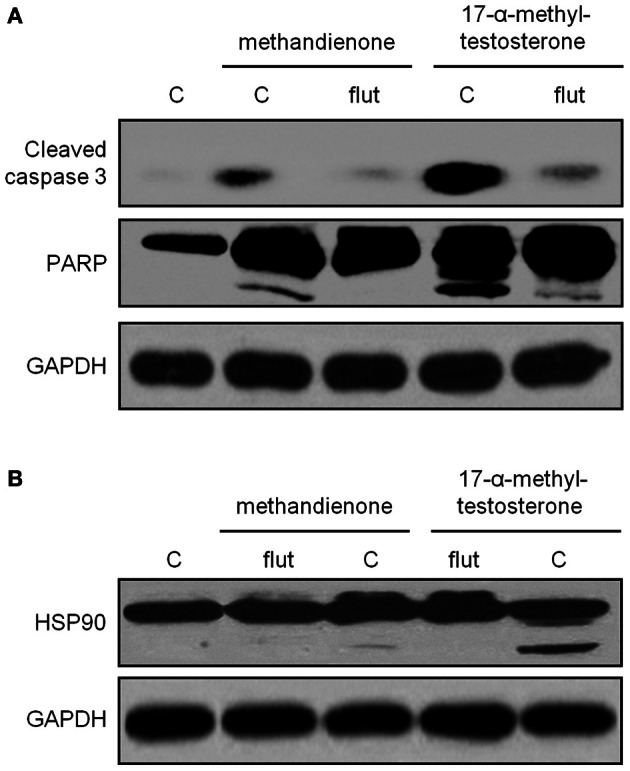
**Immunoblots for caspase 3 (*top panel*), PARP (*second panel*) (A) and Hsp90 (B) in PC12, control treated or treated for 48 h with 75 μ M methandienone and 17-α-methyltestosterone alone or with 10 μM hydroxyflutamide.** Indicators of apoptosis are decreased in the presence of hydroxyflutamide. GAPDH was used as the loading control for all blots (*bottom panels*).

To determine if apoptosis proceeds through the AR, we treated cells with methandienone and 17-α-methyltestosterone but this time in the presence of the anti-androgen hydroxyflutamide (Nguyen et al., 2007). We again observed evidence of cleavage fragments of caspase 3, PARP and Hsp90, but loss of these biochemical indicators of apoptosis when incubated with hydroxyflutamide (Figures 5A,B). This result suggests that the AR mediates the apoptotic effects of these AAS, though further experiments will be necessary to investigate this mechanism in greater detail.

### Methandienone and 17-α-methyltestosterone promote an increase in mRNA levels of neuritin

**Figure 6:**
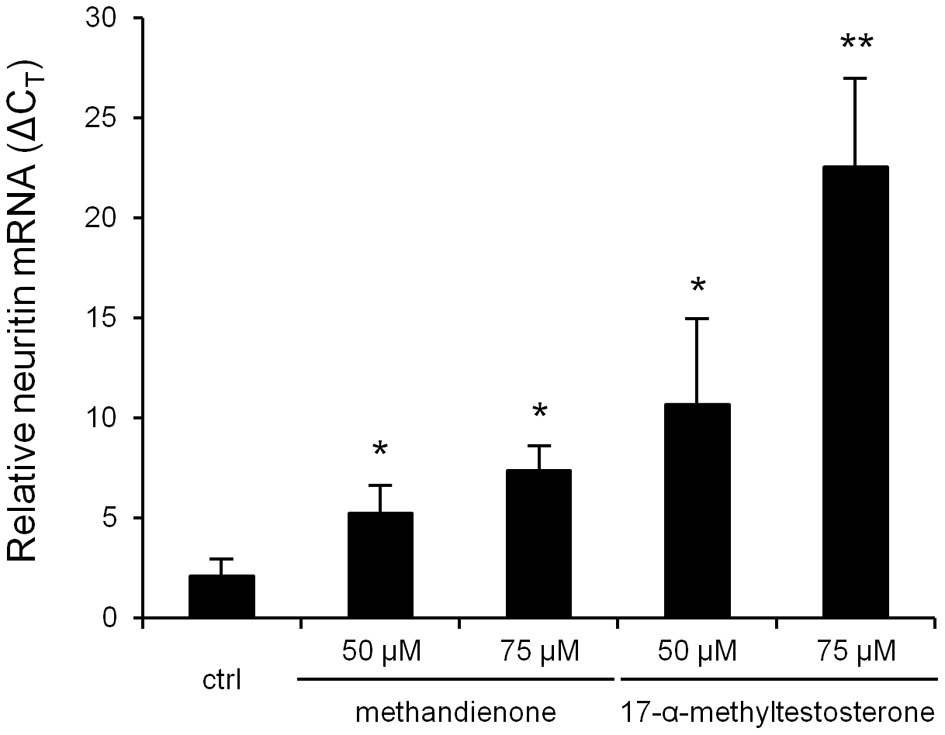
**Levels of neuritin mRNA (Δ*C*_*T*_, *Y-axis*) in PC12 treated for 24 h in the indicated concentrations of AAS, as determined by RT-qPCR normalized to GAPDH, reveal a dose-dependent increase in transcription.** Experiments were repeated three times and data expressed as mean ± SD (^*^P ≤ 0.05; ^**^P ≤ 0.01).

Neuritin is a neurotrophic factor that plays an important role in neurite growth and survival. It is known to be upregulated in damaged, stressed or ischemic neurons as they attempt to re-establish connectivity following injury (Ujike et al., 2002). Other groups have shown that certain steroids can act through the AR, which we have demonstrated is expressed in differentiated PC12 (Figure 1), to increase neuritin expression (Fargo et al., 2008). Therefore, we looked at neuritin mRNA levels in differentiated PC12 treated with increasing concentrations of methandienone and 17-α-methyltestosterone for 24 h. In these conditions, we observed a dose dependent increase in neuritin mRNA levels for both AAS tested with 17-α-methyltestosterone, which induced more cell death in the vitality assay, promoting a more dramatic upregulation of neuritin mRNA (Figure 6). These results anticipated suppression of neurite outgrowth and cell death, which were observed after 2 days of treatment, suggesting an adaptive response on the part of the cell to resist AAS toxicity.

## Discussion

There are great challenges in attempting to characterize the potential risks of neurotoxicity for environmental chemicals and pharmacological agents such as AAS. Indeed, among the innumerable commercial compounds available, a relative few have been adequately characterized for their potential effects on human health in general, and fewer still specifically tested for neurotoxicity. It is important to note that the number of studies with rigorous scientific methodology that have derived significant conclusions is small, whereas the intensity of the underground marketing and promotion of most AAS is intense, far exceeding the data supporting their use. At the same time it is important to note that non-hormonal supplements, such as vitamins, amino acids, caffeine and ephedrine often contain anabolic steroids that are not declared on the labels of the products. The most abundant steroids found in these supplements are methandienone and 17-α-methyltestosterone.

It has been suggested that tests based on a variety of *in vitro* assessments may be useful as a screen to identify neurotoxic substances. We employed several cell biological and biochemical techniques to demonstrate that methandienone and 17-α-methyltestosterone were damaging neuron-like differentiated PC12 cells through inhibition of neurite networks and apoptosis, suggesting that administration of these AAS for the purpose of enhancing athletic performance could put the user at risk for even more potentially harmful side effects in addition than the better known effects on the endocrine, musculo-skeletal, and reproductive systems.

The development of the CNS involves coordinated gene expression and an ordered initiation of specific cellular events regulating proliferation, differentiation, cell migration, neurite outgrowth, synapse formation, myelination, and programmed cell death. Theoretically, chemically mediated disruption of one or more of these events could potentially impair CNS development or function (Barone et al., 2000). Neurite outgrowth in particular involves many of these processes, including the differentiation of precursor cells to a terminal neuronal phenotype and the initiation and development of broad, sheet-like extensions (lamellipodia) which subsequently condense into short cytoplasmic processes capable of communicating with neighboring cells through synaptogenesis (Craig and Banker, 1994). Alterations in neuronal signaling and synapse formation likely contribute to the outward psychological and behavioral manifestations of steroid use (Penatti et al., 2009, 2011; Oberlander and Henderson, 2012). However, while synapse signaling is one of the first processes to be affected in the premature aging model, for example, neuron degeneration is known to underly many behavior disorders (de Graaf et al., 2013). Therefore, we limited our study to morphology and cell death utilizing the PC12 cell model. PC12s are a well characterized *in vitro* model for evaluation of chemical neurotoxicity because they differentiate into a sympathetic-like neurons and develop extensive neuritic projections following exposure to NGF (Fujita et al., 1989; Vaudry et al., 2002; Radio and Mundy, 2008). Many studies have used PC12 cells to evaluate the effect of environmental chemicals on neurite outgrowth (Chan and Quik, 1993; Das and Barone, 1999; Lein et al., 2000; Crumpton et al., 2001; Parran et al., 2003). Using this model, both neuroprotective (Fargo et al., 2008) and neurodegenerative effects (Radio and Mundy, 2008) of androgens have been described. In the present study, we investigated the effects of supraphysiological amounts of two different AAS, methandienone and 17-α-methyltestosterone, and observed dramatic changes in the sympathetic neuron-like PC12 cell line.

The concentrations of steroids used in our experiments are comparable to that used in other studies examining different compounds considered to be performance-enhancing. For example, in Duranti's study, rat L6C5 and mouse C2C12 skeletal muscle cells were treated with up to 20 μM of the β_2_-adrenergic receptor agonist salmeterol at a concentration of 1 × 10^4^ cells per square centimeter, conditions comparable to our AAS experiments (Duranti et al., 2011). Indeed, the initial concentration of cells per square centimeter used in our studies is the minimum required to allow neuron-like cells to create a neurite network. Our results demonstrate that methandienone and 17-α-methyltestosterone are detrimental to the development of neuron-like characteristics in PC12 cells, and therefore could be considered to have a high risk of neurotoxicity *in vivo.* As a result, these compounds will likely require further testing.

AAS that previously had been shown to damage neurite networks in PC12 cells have also been shown to activate the apoptotic pathway. Having detected evidence of toxicity in the neurite outgrowth assay, we then wanted to determine if the continued exposure of differentiated PC12 to AAS might induce cell death. Therefore, we examined PC12 cells in a vitality assay and observed evidence of permeability to an acridine orange/ethidium bromide mixture, and hence loss of membrane integrity and cell death, following exposure to methandienone and 17-α-methyltestosterone, with the latter showing slightly more cell death than the former. To determine the nature of cell death, we investigated phospho-ERK, a marker of ell vitality and survival, and several components of the apoptotic pathway. Phosphorylation of ERK was reduced upon exposure to androsterone, nandrolone, methandienone and 17-α-methyltestosterone. Caspase 3 is one of the key executioners of apoptosis, engaging in the proteolytic cleavage of many key proteins such as the nuclear enzyme PARP. Indeed, the finding that caspase 3 is expressed in PC12 suggests a role for this protease in PC12 cell death (Haviv et al., 1997). We detected the appearance of the cleaved and hence active form of caspase 3, along with the cleaved form of PARP, in an immunoblot of PC12 exposed to all AAS tested, suggesting that apoptosis might be a generalized response to high concentrations of steroids. When using methandienone and 17-α-methyltestosterone, dose dependent increases in these indicators of apoptosis were seen, with a slightly more dramatic response for the same given concentrations of 17-α-methyltestosterone. Taken together, these results demonstrate that AAS induce apoptosis in neuritized PC12.

We noted that the effects on cells following AAS treatment were delayed, suggesting that these hormones might exert their effects by acting on AR-mediated genomic pathways. Though previous efforts have failed to detect the AR receptor in PC12 cells by RT-PCR (Nguyen et al., 2005), we did in fact demonstrate AR receptor expression in both rat brain (as a control) and in neuron-like PC12 cells growing in culture, supporting the findings of other investigators (Gehlhaus et al., 2007; Meyer et al., 2009) and in a distribution pattern similar to that identified through confocal microscopy in prior publications (Caraci et al., 2011). While androgens can exert rapid non-genomic effects involving induction of second messenger signal transduction cascades, these lipophilic hormones also induce AR dimerization and translocation into the nucleus where the complex engages in direct protein-protein interaction with transcriptional co-regulators at cognate palindromic response elements to promote the expression of target genes. The presence of AR in PC12 and the delayed responses we observed support a possible genomic influence on cell viability for the AAS (Imperlini et al., 2010). Therefore, to investigate if the mechanism proceeds through AR stimulation, we treated cells with methandienone and 17-α-methyltestosterone alone or with the anti-androgen hydroxyflutamide. Loss of indicators of apoptosis in the presence of hydroxyflutamide suggest that AAS-mediated apoptosis proceeds through the AR and might therefore alter gene transcription (Heinlein and Chang, 2002). However, further detailed study is required.

Hsp90 is a molecular chaperone responsible for controlling numerous signaling pathways in the cell (Bishop et al., 2007). It is essentially a protective protein, keeping pro-apoptotic factors in an inert state while simultaneously regulating anti-apoptotic proteins (Lewis et al., 2000). Other studies have shown that cleavage of Hsp90 correlates with activation of apoptosis through both the intrinsic or mitochondrial-mediated death pathway as well as through extrinsic or receptor-mediated pathways (Arya et al., 2007; Chen et al., 2009). Our studies support these conclusions with the observation of the appearance of a cleaved fragment of Hsp90 following treatment of PC12 cells with methandienone and 17-α-methyltestosterone.

We also observed a dose-dependent increase in neuritin mRNA levels in PC12 cells treated with methandienone and 17-α-methyltestosterone, which to our knowledge is the first *in vitro* evidence of androgen-mediated regulation of neuritin mRNA levels in PC12 cells. Neuritin is a gene that regulates androgen-induced (and AR-dependent) neurite outgrowth in motor neurons, usually in response to an injury. In cultured cells, recombinant neuritin protein enhances neurite extension and branching (Naeve et al., 1997). However, unlike in these prior reports, we show that neuritin increases occurred prior to the visible manifestations of loss of neurite formation and cell death. This discrepancy could be attributable to the concentrations of AAS used in our study, which were an attempt to approximate the amounts used by athletes to enhance performance, or differences in cell type and experimental conditions. We regarded increased production of this neurotrophic factor as a response to stress and an attempt at cell survival, as continued exposure to methandienone and 17-α-methyltestosterone eventually induced apoptosis. In contrast to our other findings, methandienone elicited a less significant response in this assay (a lower increase in neuritin mRNA) when compared to administration of the same amounts of 17-α-methyltestosterone. If 17-α-methyltestosterone truly is more harmful to neuron-like cells than methandienone, as suggested by the vitality assay and apoptosis immunoblots, then it could have promoted higher levels of neuritin transcription as an adaptive response to resist the greater toxicity for this AAS. The differences we observed in PC12 responses to methandienone and 17-α-methyltestosterone as well as protein expression patterns warrant further investigation.

Here we show that at high concentrations, methandienone and 17-α-methyltestosterone exert detrimental effects on differentiated PC12 cells expressing AR, including inhibition of neurite network maintenance, induction of cell death through apoptosis and cleavage of the protective chaperone protein Hsp90. Between these two compounds we noted greater cell death and higher neuritin transcription in PC12 in response to 17-α-methyltestosterone treatment, supporting the belief that this AAS is the more toxic to neuron-like cells of the two compounds tested. These findings will be pursued in future investigations but currently suggest another potentially harmful physiological effect in the abuse of steroids, that of CNS toxicity.

## Authors contributions

John R. Basile provided protocols and support for immunohistochemistry and immunoblot experiments and helped draft the manuscript. Nada O. Binmadi assisted in immunohistochemistry, immunocytochemistry and neurite outgrowth experiments. Hua Zhou and Ying-Hua Yang performed the RT-PCR for neuritin. Antonio Paoli helped plan out experiments and draft the manuscript. Patrizia Proia conceived and designed the study, performed the vitality assays and helped draft the manuscript. All authors read and approved the final manuscript.

### Conflict of interest statement

The authors declare that the research was conducted in the absence of any commercial or financial relationships that could be construed as a potential conflict of interest.

## Abbreviations

AAS: anabolic-androgenic steroids
CNS: central nervous system
AR: androgen receptor
PARP: poly (adenosine diphosphate [ADP]-ribose) polymerase
Hsp: heat shock protein
NGF: nerve growth factor
PC12: pheochromocytoma 12 cells
EB: Ethidium bromide
AO: Acridine orange.
